# Psychosocial and personal predisposing factors of frostbite injury and associated amputation: a systematic review

**DOI:** 10.1186/s40621-024-00546-w

**Published:** 2024-11-07

**Authors:** Samuel Kwaku Essien, Batholomew Chireh, Chantee Steinberg, Phinehas Omondi, Audrey Zucker-Levin

**Affiliations:** 1 Department of Health Sciences, Lakehead University, 955 Oliver Road Thunder Bay, Thunder Bay, ON P7B 5E1 Canada; 2 EPID@Work (Enhancing the Prevention of Injury & Disability @ Work) Research Institute, Lakehead University, Thunder Bay, ON Canada; 3 School of Rehabilitation Science, University of Saskatchewan, Saskatoon, SK Canada

**Keywords:** Frostbite, Amputation, Psychosocial factors, Personal factors, Injuries, Limb loss, Cold regions

## Abstract

**Objective:**

To date, systematic reviews of frostbite injuries predominantly focus on the treatment of frostbite, which narrows the scope of prevention and disregards the impact of frostbite-related predisposing factors. Comprehensively synthesizing relevant evidence to understand the psychosocial and personal predisposing factors to frostbite injury and related amputation would provide new insight into frostbite injury prevention. This review investigated the psychosocial and personal predisposing factors of frostbite injury and associated amputation.

**Methods:**

Databases, including Embase, PubMed, and PsycINFO, were systematically searched for relevant studies. Two independent reviewers performed the screening, data extraction, and quality assessment. Inclusion criteria were studies that reported cold injury, predisposing factors for frostbite injury or related amputations, and assessed the relationship between a predisposing factor and the frostbite injury or amputation outcome based on a descriptive or inferential test.

**Results:**

Thirty-six (36) studies met the inclusion criteria; 29 reported on both frostbite injury and amputations, and seven reported on only frostbite injury. Six psychosocial predisposing factors were observed in 28 out of the 36 studies reviewed, which included people experiencing homelessness, low socioeconomic status, alcohol intoxication/abuse, smoking, psychiatric disorders, and substance use. Personal predisposing factors identified included inadequate/improper winter clothing, delay in seeking medical care, and lack of knowledge of how to deal with the cold.

**Conclusions:**

While it is crucial to allocate additional resources and research toward improving the treatment of individuals affected by frostbite injuries and associated limb loss, it is equally important to direct efforts toward addressing the psychosocial and personal predisposing factors that predispose individuals to these injuries and amputations.

## Background

Geographical jurisdiction can expose individuals to unique injuries and medical conditions, including frostbite injuries and related amputation due to tissue freezing and damage (Carceller et al. 2019; Murphy et al. (2000); Basit 2024). The novelty of this injury is that it predominantly occurs in regions with colder climates, especially during the winter season (Basit 2024), more specifically in temperatures below − 0.55 C (31 F) (National Health Service (NHS) 2023; Regli et al. 2021). The resultant injuries from the freezing conditions may range from mild form (frostnip) to a more severe gangrenous condition necessitating digit or limb amputation (Gupta et al. 2021). Many survivors of frostbite injury are subject to long-term consequences, including vasomotor disturbances, chronic pain, arthritis and mobility issues (Regli et al. 2021), which often negatively impact quality of life (Gao et al. 2021).

Although frostbite-associated amputation is uncommon compared to amputation caused by vascular disease, trauma, and cancer (Imam et al. 2017), the rising incidence in countries with colder climates, such as Canada (Mulcahy 2024; Hoye 2024; Whitfield 2024), raises concern about the need to identify major psychosocial and personal predisposing factors of frostbite injury and associated amputation and determine whether these factors align with findings across geographical regions in the world. Recent reports from the Canadian provinces of Alberta, Saskatchewan, and Manitoba identified a decade-high number of frostbite-associated amputations (91, 18, and 19 cases, respectively) in the 2021-22 fiscal year (Mulcahy 2024; Hoye 2024; Whitfield 2024). Similarly, a 2022 report from Alaska, United States, revealed that at least 262 cases of frostbite-associated amputation were recorded over the preceding five years (Boots 2024). Frostbite injury may result from several outdoor activities that predominately occur in cold weather conditions, including work exposures (Borud et al. 2018), inadequate preparation for outdoor exposure (Hobson B, The Canadian Press 2023), and leisure activities (Carceller et al. 2019; Eun 2023). Moreover, media reports have linked frostbite-associated amputation to cross-border immigration and whiteouts during winter (Hobson B, The Canadian Press 2023; George J, Nunatsiaq News 2024). For example, cross-border immigration through the United States-Canada border in Manitoba in the winter led to frostbite-associated finger amputations (Hobson B, The Canadian Press 2023). Also, a whiteout in Cambridge Bay, Nunavut, resulted in severe frostbite-associated bilateral hand amputation (George J, Nunatsiaq News 2024).

Despite these known factors from different reports, no systematic review has been conducted to comprehensively synthesize the global literature for psychosocial and personal factors, such as homelessness, substance use, psychiatric disorder and improper winter clothing (Zhang et al. 2022), that predispose individuals to frostbite injury and associated amputation. To date, systematic reviews in this line of research predominantly focus on the treatments of frostbite (Hutchison et al. 2019; Drinane et al. 2019); although imperative, reviews that comprehensively synthesize relevant evidence of psychosocial and personal predisposing factors for frostbite injury and associated amputation can serve as primordial prevention and help decrease the incidence of frostbite and associated amputation.

Furthermore, psychosocial and personal predisposing factors for frostbite-related amputation may differ among populations (Ghumman et al. 2019), but how these factors influence frostbite injury across the world’s cold regions remains unclear. Studies published in Canada found psychiatric disorders as a predisposing factor for frostbite injuries and related amputation (Ghumman et al. 2019; Urschel 1990). However, Endorf et al.’s study on frostbite patients in the United States noted in their multivariate analysis that other psychiatric disorder diagnosis was not associated with amputation (Endorf et al. 2022a).

The ambiguity in reported predisposing factors further highlights the need for comprehensive information on frostbite injury and associated amputation. Identifying key psychosocial and personal predisposing factors would allow for focused and targeted interventions/preventions to help alter the rising incidence of frostbite injury and associated amputation. Therefore, this systematic review aims to identify psychosocial and personal predisposing factors associated with frostbite injury and frostbite-associated limb amputation across geographical locations.

## Methods

### Search strategy

This review adhered to the PRISMA approach (Liberati et al. 2009). It utilized several electronic databases that index literature from medical science, including OVID Medline, Embase, PubMed, Cochrane Library, Cumulative Index to Nursing and Allied Health Literature (CINAHL), SCOPUS and PsycINFO. All databases were searched from their respective start dates until the 25th September 2024. We used a three-stage search strategy to identify eligible studies. At the first stage, we searched for keywords reflecting amputation (“amputation” OR limb loss* OR “limb amputation” OR “amputee” OR “upper limb amputation” OR “lower limb amputation” OR “amput*”). In the second stage, we searched for common words for frostbite (“frostbite” OR “frostbite injury” OR “frostbites” OR “frostbit*”). In the third stage, we combined step I AND step II AND (psychosocial OR environmental OR determinants OR frostbite amputation OR predisposing factors of observational studies) for study literature retrieval. We also manually reviewed the reference lists of the identified studies to discover any additional relevant studies.

## Inclusion and exclusion criteria

The inclusion of articles in the study was determined based on studies conforming to the following inclusion criteria: (1) studies must report a cold injury because of exposure to freezing temperatures, which results in amputation or frostbite injury; (2) focus on determining psychosocial and personal predisposing factors for frostbite-related amputation (either lower or upper extremity) or frostbite injuries; (3) report an association between psychosocial and personal predisposing factor and frostbite-related amputation or frostbite injuries backed by statistical inferential test (e.g., p-value, risk ratio or odds) or describe the relationship using case reports or case series; (4) confined to English-language publications; (5) No restriction on date of publication, study design, age, sex or geographical location.

## Data screening and extraction

**Fig. 1:**
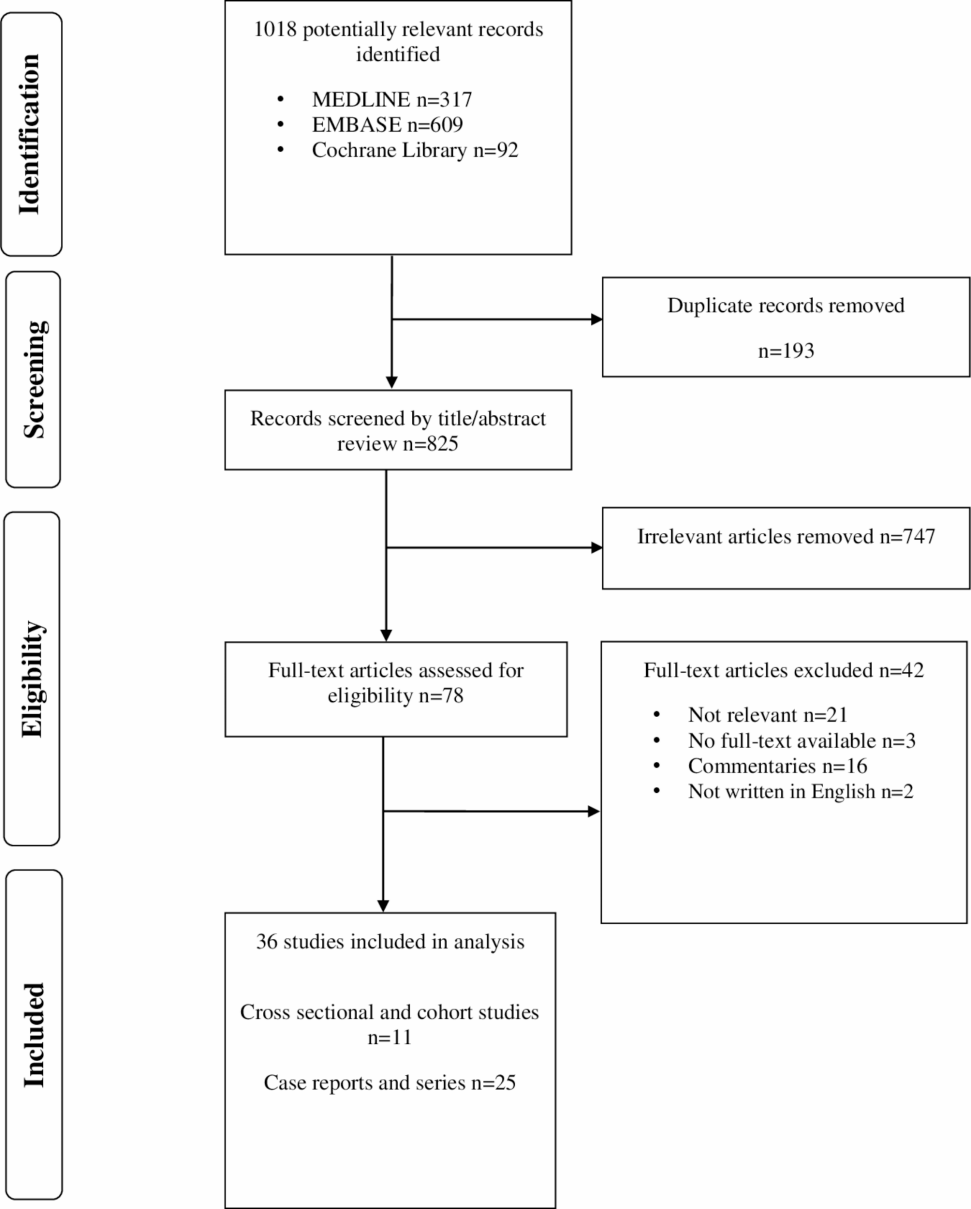
PRISMA flowchart of psychosocial and personal predisposing factors of frostbite injury and associated amputation

Screening and selection of all identified articles were performed by title, abstract, and full text, which was completed by (CS) and (PO). Data extracted from the selected studies included population and study characteristics (demographics, geographical locations, comorbidities, study design, study samples, and parts of the body affected by the frostbite-related amputation, including level and types of amputation); predisposing factors, severity of frostbite injury into superficial (first and second degree) and deep (third and fourth degree) (Fabian et al. 2017) and results of inferential tests that quantified the association between predisposing factors and frostbite-related amputation. Two reviewers (CS) and (PO) independently extracted data using an Excel spreadsheet. In instances where differences in opinion arose, the reviewers engaged in discussions, and a third reviewer came in as a tiebreaker before resolving. Regular meetings were also held throughout the screening and selection process to discuss outstanding concerns. Figure 1 below provides details on the article selection process (PRISMA flowchart).

## Selection of articles

The initial search yielded 1018 abstracts, and after duplicates were removed, 825 articles’ titles and abstracts were screened. A total of 78 full-text articles were screened and after the full-text screening, 42 were excluded, leaving 36 articles included in the review. Attributes including sample size, age, sex, study design, predisposing factors, severity of frostbite injury/degree of frostbite injury and extremity amputation are presented in Table 1 for studies that report on frostbite-related amputation; Table 2 includes the same attributes excluding extremity amputated for studies that report on frostbite injury only; Table 3 contain key and significant findings associated with frostbite-related amputation, and Table 4 shows key and significant findings associated with frostbite-related injury alone. Out of the 36 studies identified, 21 studies were case series and four case reports, providing a detailed description of cases of frostbite injuries and associated limb loss; only four studies examined predisposing factors of frostbite-related amputation at a specific point in time (i.e., cross-sectional study) while six studies followed and observed the same individuals over a period (i.e., retrospective cohort studies) and one used prospective case-control study.

**Table 1:** Summary of studies that include frostbite and frostbite-related limb amputation attributes

Author(s), year, country	Study sample size	Age years (mean/median)	Sex (M/F)	Study design	Degree of frostbite injury	Predisposing factors	Limb loss outcome/amputation extremity^*^
Antti-poikka et al., 1990, Finland	9	49	9/0	RCS	Deep	**Psychosocial** -Alcohol intoxication/abuse	UE & LE
Boles et al., 2018, Canada	47	15	24/23	RCS	Superficial and deep	**Psychosocial** -Alcohol intoxication/abuse -Cigarette use -Marijuana use -Depression -Unsupervised care	UE & LE
Brandstrom et al., 2014, Sweden	244	60	164/80	RCS	Superficial and deep	**Psychosocial** -Alcohol intoxication/abuse	NS
Carceller et al., 2019, Spain	92	33.1	74/18	RCS	Deep	**Personal (amputation-specific)** -Delay before receiving medical attention -Time before rewarming	NS
Cauchy et al., 2001, France	70	31.9	63/7	RCS	Superficial and deep	**Psychosocial** -Alcohol intoxication/abuse -Psychiatric disorders **Personal** -Inadequate or improper winter clothing	UE & LE
Detanac et al., 2022, Serbia	1	40	1/0	CR	Deep	**Psychosocial** -Homelessness -Psychiatric disorders **Personal** -Inadequate or improper winter clothing	NS
Endorf et al., 2022a, USA	148	42.4	120/28	RC	Superficial and deep	**Psychosocial** -Alcohol intoxication/abuse -Homelessness -Substance use	NS
Endorf and Nygaard, 2022b, USA	42,810	49.2	29,585/13,225	RC	Deep	**Psychosocial** -Homelessness	UE & LE
Endorf and Nygaard, 2021a, USA	1065	51.2	855/210	RC	Superficial and deep	**Psychosocial** -Alcohol intoxication/abuse -Homelessness -Psychiatric disorders -Substance use	UE & LE
Endorf and Nygaard, 2021b, USA	1617	51.3	1301/316	RC	Superficial and deep	**Psychosocial** -Alcohol intoxication/abuse -Homelessness -Psychiatric disorders -Substance use	UE & LE
Fabian et al., 2017, Canada	265	40.5	196/69	RC	Superficial and deep	**Psychosocial** -Alcohol intoxication/abuse -Psychiatric disorders **Personal** -Inadequate or improper winter clothing	UE & LE
Hashmi et al., 1998, Pakistan	1500	27	1500/0	RCS	Superficial and deep	**Personal** -Inadequate or improper winter clothing	NS
Jovic et al., 2019, Serbia	24	49	21/3	RCS	Superficial and deep	**Psychosocial** -Alcohol intoxication/abuse -Homelessness -Psychiatric disorders -Smoking	UE & LE
Kloeters et al., 2011, Germany	1	19	1/0	CR	Deep	**Unexpected Event** Delay before receiving medical care after a car accident	UE & LE
Koljonen et al., 2004, Finland	42	42.5	36/6	RCS	Superficial and deep	**Psychosocial** -Alcohol intoxication/abuse -Psychiatric disorders	UE & LE
Lindford et al., 2017, Finland	20	51.1	16/4	RC	Superficial and deep	**Psychosocial** -Alcohol intoxication/abuse -Psychiatric disorders -Smoking -Substance use	UE & LE
Lorentzen and Penninga, 2018, Greenland	6	43.8	5/1	CR	Superficial and deep	**Psychosocial** -Alcohol intoxication/abuse -Psychiatric disorders **Personal** -Inadequate or improper winter clothing	UE
Miller et al., 1980, Canada	101	NS	NS	RCS	Superficial and deep	**Psychosocial** -Alcohol intoxication/abuse	UE & LE
Nygaard et al., 2017, USA	73	42	58/15	RCS	Deep	**Psychosocial** -Alcohol intoxication/abuse -Homelessness -Substance use -Psychiatric disorders **Personal (amputation-specific)** -Delay before receiving medical attention -Time before rewarming	UE & LE
Poole et al., 2021, Canada	22	39	17/5	RCS	Superficial and deep	**Psychosocial** -Alcohol intoxication/abuse	UE & LE
Schellenberg et al., 2020, USA	241	44	184/57	RCS	NS	**Personal (amputation-specific)** -Higher admission heart rate	UE & LE
Su et al., 2015, China	568	NS	NS	RCS	Superficial and deep	**Psychosocial** -Alcohol intoxication/abuse -Psychiatric disorders	UE & LE
Tavri et al., 2016, USA	13	33.4	11/2	RCS	Deep	**Psychosocial** -Alcohol intoxication/abuse -Homelessness -Tobacco use	UE & LE
Tran et al., 2022, USA	54	NS	38/16	RCS	NS	**Psychosocial** -Alcohol intoxication/abuse -Homelessness -Psychiatric disorders -Substance use	UE & LE
Urschel 1990, Canada	79	NS	NS	RCS	NS	**Psychosocial** -Alcohol intoxication/abuse -Psychiatric disorders **Personal (amputation-specific)** -Delay before receiving medical attention	NS
Valnicek et al., 1993, Canada	125	41	97/28	RCS	Superficial and deep	**Personal** -Inadequate or improper winter clothing **Personal (amputation-specific)** -Delay before receiving medical attention	UE & LE
Wani et al., 2008, India	1	15	0/1	CR	Deep	**Psychosocial** -Low socioeconomic status **Personal** -Delay before receiving medical attention -Inadequate or improper winter clothing -Lack of knowledge of how to deal with cold weather	LE
Zhang et al., 2022, China	36	51.5	30/6	RCS	Deep	**Psychosocial** -Alcohol intoxication/abuse -Homelessness -Psychiatric disorders -Smoking -Substance use **Personal** -Inadequate or improper winter clothing	LE
Zhao et al., 2020, China	156	43.7	128/28	RCS	Deep	**Psychosocial** -Alcohol intoxication/abuse -Smoking **Personal (amputation specific)** -Delay in medical treatment -Longer time period before rewarming	UE & LE

Upper Extremity (UE), Lower Extremity (LE), Extremity not specified (NS)^*^

Retrospective Case-series (RCS), Retrospective Cohort (RC), Case Report(s) (CR), Cross-Sectional (CS)

Prospective Case-Control (PCC)*

**Table 2:** Summary of studies that include frostbite injury alone (no amputation attributes)

Author(s), year, country	Study sample size	Age years (mean)	Sex (M/F)	Study design	Degree of frostbite injury	Predisposing factors
Ervasti et al., 2004, Finland	5893	20	5893/0	CS	Superficial and deep	**Psychosocial** -Smoking
Ghumman et al., 2019, Canada	22	40	18/4	RCS	Superficial and deep	**Psychosocial** -Alcohol intoxication/abuse -Psychiatric disorders -Substance use
Hanko et al., 2022, USA	41	NS	29/12	RCS	Superficial and deep	**Psychosocial** -Homelessness -Psychiatric disorders -Substance use
Harirchi et al., 2005, Iran	637	29.6	NS	CS	Superficial and deep	**Personal** -Inappropriate clothing -Lack or incorrect use of equipment -Lack of knowledge of how to deal with cold weather
Lehmuskallio et al., 1995, Finland	913	19	913/0	PCC	Superficial and deep	**Personal** -Inadequate or improper winter clothing
Makinen et al., 2009, Finland	13,713	NS	NS	CS	Superficial and deep	**Psychosocial** -Alcohol intoxication/abuse -Psychiatric disorders
Masood et al., 2008, Pakistan	130	29.06	130/0	CS	Superficial	**Personal** -Lack or incorrect use of equipment -Lack of knowledge of how to deal with cold weather

**Table 3:** Summary of studies that include frostbite-related amputation

Author(s), year, country	Main findings
Antti-poikka et al., 1990, Finland	▪ 9 patient cases were included in this retrospective case-series ▪ 7 patients required frostbite-related amputation due to alcohol intoxication, with 6 cases (66%) occurring in patients with histories of chronic alcohol abuse ▪ 2 patients had psychological disorder (schizophrenia) ▪ The major predisposing factor for frostbite in this population was acute and/or chronic alcohol consumption
Boles et al., 2018, Canada	▪ 47 patients ranging from 1 to 17 years old met the inclusion criteria for this retrospective case series. ▪ Two important factors related to frostbite injury were identified: lack of supervision and intoxication. ▪ Frequently documented risk activities included the use of alcohol (*n* = 25, 53%), cigarettes (*n* = 16, 34%), marijuana (*n* = 11, 23%), and symptoms of depression with or without suicidal ideations (*n* = 15, 32%).
Brandstrom et al., 2014, Sweden	▪ 244 patient cases were included in this retrospective case-series ▪ Alcohol consumption was an associated factor for frostbite injury ▪ Higher rates of frostbite occurred among males
Carceller et al., 2019, Spain	▪ 92 patient cases were included in this retrospective case-series ▪ There were no differences found in amputation risk regarding age, gender, smoking, or occupation among patients with frostbite injuries ▪ Time before rewarming and delay before receiving medical attention for the frostbite injury are both risk factors for amputation
Cauchy et al., 2001, France	▪ 70 patient cases were included in this retrospective case-series ▪ Two cases were related to alcohol intake or a psychiatric disorder ▪ It was found that in most cases, frostbite resulted from inadequate protection/ winter clothing (due to poor knowledge of the terrain, dehydration and/ or exhaustion)
Detanac et al., 2022, Serbia	▪ This study describes the case report of a 40-year-old black male in Serbia who walked for a long period of time in cold winter weather and twice left the hospital against medical advice. ▪ The occurrence of frostbite is more frequent in the homeless, people who abuse alcohol, and those with a psychiatric disorder
Endorf et al., 2022a, USA	▪ 148 people were included in this retrospective cohort study ▪ 40 people (18%) required amputation ▪ Substance and/or alcohol use independently predicted frostbite-related limb amputation ▪ Substance use disorders (Odds Ratio (OR): 3.19; 95%CI 1.15–8.81) and homelessness (OR: 5.40; 95%CI 1.53–19.09) were independent predictors of unplanned hospital re-admission
Endorf and Nygaard, 2022b, USA	▪ This retrospective cohort study included 42,810 people ▪ During non-elective primary admission, homelessness (OR: 1.81; 95%CI 1.31–2.49) was associated with frostbite-related limb amputation ▪ Although substance and alcohol use were prevalent within the population with frostbite injury, there was no association of these factors with an increase in amputation ▪ Surprisingly, psychiatric disorders (OR:0.64; 95%CI 0.43–0.94) were not associated with frostbite-related limb amputation
Endorf and Nygaard 2021a, USA	▪ This retrospective cohort study involved 1065 patients, 29% of whom sustained frostbite injuries that resulted in at least one amputation ▪ The social factors associated with frostbite injury were homelessness, psychiatric disorders, and mental or behavioural disorders resulting from substance use
Endorf and Nygaard 2021b, USA	▪ This retrospective cohort study included 1617 people ▪ Approximately 34.6% of the frostbite injuries were related to homelessness, 35.2% were related to a mental health diagnosis, and 80.9% were related to a substance and/or alcohol use diagnosis ▪ Significant factors associated with a higher risk of frostbite-related limb amputation include Black race (OR: 1.71; 95%CI 1.22–2.39), homelessness (OR: 1.62; 95%CI 1.20–2.20), and male gender.
Fabian et al., 2017, Canada	▪ 265 people were included in this retrospective cohort study ▪ Prevalent predisposing factors for frostbite injuries included inadequate clothing/footwear (32.1%), alcohol abuse (15.1%), and psychiatric illness (20.8%) ▪ Comparing patients with deep frostbite to those with superficial frostbite, smoking was more prevalent, and patients were older (p-value < 0.001)
Hashmi et al., 1998, Pakistan	▪ 1500 patient cases were included in this retrospective case-series ▪ Frostbite injuries to the feet were most prevalent (64%), with substandard or damaged footwear being a likely factor ▪ Patients with proper education were able to recognize early symptoms of frostbite and thus receive treatment faster, lessening the degree of injury
Jovic et al., 2019, Serbia	▪ 24 patient cases were included in this retrospective case-series ▪ Of the patients treated for frostbite, 58% were chronic alcohol consumers, 46% were long-term smokers, 33% suffered from psychiatric disorder, and 13% were homeless ▪ Most patients were male (88%)
Kloeters et al., 2011, Germany	▪ This case report details the case of a 19-year-old man involved in a car accident on an abandoned road in rural Germany, in which he fell unconscious during cold Winter temperatures ▪ The main suggestion of this report is that early initiation of rescue triage is paramount to ensure devastating injuries do not progress
Koljonen et al., 2004, Finland	▪ 42 patient cases were included in this retrospective case-series ▪ Of the 42 patients, 92 frostbite injuries were recorded ▪ 21 (50%) of the patients did not have surgery, 4 (10%) of the patients had minor surgery, and 17 (40%) had major surgery ▪ At the time of the frostbite injury, 25 (60%) of the patients were intoxicated by alcohol, 6 (14%) patients were homeless, and 14 (35%) had a mental illness
Lindford et al., 2017, Finland	▪ 20 patient cases treated for frosbite injury were included in this retrospective case-series ▪ 75% of the patient cases included societal risk factors, which included alcohol abuse, drug abuse, psychiatric illness, and smoking ▪ Of the societal risk factors, alcohol abuse was the most common and was found in 9 (45%) cases
Lorentzen and Penninga, 2018, Greenland	▪ 6 patient case reports were included in this study ▪ Of the total number of 6 patients, only 1 patient (16.7%) required amputation after a frostbite injury ▪ Most patients were males (83.3%) ▪ The cases illustrate some of the known risk factors for frostbite injuries, such as alcohol consumption and inadequate winter clothing (e.g. improper, ill-fitting or missing footwear, lack of gloves)
Miller et al., 1980, Canada	▪ 101 patient cases were included in this retrospective case series, with 66 receiving treatments for frostbite ▪ Amputation was required for one-third of patients treated in Saskatoon (representing 22 out of the 66 patients receiving treatment for frostbite). ▪ 59% of the frostbite injury cases included alcohol consumption as a contributing factor
Nygaard et al., 2017, USA	▪ 73 patient cases were included in this retrospective case series ▪ Most patients were males (80%) ▪ Factors associated with increased risk of frostbite injury included: homelessness (22%), alcohol abuse (69%), drug abuse (29%), and psychiatric illness (38%)
Poole et al., 2021, Canada	▪ 22 patient cases were included in this retrospective case series ▪ Alcohol use was a factor in 27% of cases, and was found to result in more severe frostbite injury
Schellenberg et al., 2020, USA	▪ 241 patient cases were included in this retrospective case-series ▪ 42% of patients with frostbite injury required ICU admission and 5% required amputation ▪ Patients admitted to the ICU had a greater need for amputation compared to those not admitted (9% vs. 4%, p-value = 0.04) ▪ Higher admission heart rate was an independent predictor of amputation (p-value = 0.013).
Su et al., 2015, China	▪ 568 patient cases were included in this retrospective case-series ▪ Patients who were admitted less than 1 day after sustaining frostbite injury had lower amputation rates than those whose admission time was greater than 1 day (p-value < 0.001) ▪ Amputation rates of patients who sustained frostbite injuries while consuming alcohol, experiencing psychiatric illness, and trauma (including traffic accidents) were higher than those injuries caused by improper protection and going astray (p-value < 0.01)
Tavri et al., 2016, USA	▪ 13 patient cases were included in this retrospective case-series ▪ Out of the 13 patients, 61.5% (8 patients) did not require amputation after frostbite injury, while 38.5% (5 patients) underwent amputation ▪ The characteristic predisposing risk factors of the patients included alcohol intoxication (76.9%), tobacco use (53.8%), homelessness (12.4%)
Tran et al., 2022, USA	▪ 54 patient cases were included in this retrospective case-series ▪ Of the 54 patients with frostbite, the characteristics are as follows: male (70%), substance use disorder (50%), and homeless (14%). ▪ There were no significant differences in the number of surgeries or amputations required among these groups of patients; however, patients with a positive substance use disorder screening, psychiatric disorders, active drug use, and homelessness were more likely to be readmitted for wound infections of gangrene progression. ▪ Psychiatric disorders were predictive of requiring additional surgeries (p-value = 0.02) and longer hospital stays (p-value = 0.046).
Urschel, J., 1990, Canada	▪ 79 patient cases were included in this retrospective case-series ▪ At the time of frostbite injury, it was found that 53% of the patients were under the influence of alcohol and 16% had a psychiatric illness ▪ A delay in seeking medical attention was found to be a factor associated with a poor outcome (amputation)
Valnicek et al., 1993, Canada	▪ 125 patient cases were included in this retrospective case-series ▪ Factors that did not show a statistical correlation with the need for amputation include history of alcohol intoxication, psychiatric illness, history of smoking, and age ▪ Significant factors associated with frostbite-related amputation included duration of cold exposure, lack of proper attire, location of injury in the wilderness, presence of wound infection, and delay in seeking treatment greater than 1 day
Wani et al., 2008, India	▪ This case report presents the case of a 15-year-old female patient of low socioeconomic status who suffered frostbite to her feet while dwelling outdoors in the snow to collect water and timber for her household. ▪ She sustained fourth-degree frostbite injuries, which sequentially led to autoamputation of the distal portion of her feet over time. ▪ She had married in a low socio-economic stratum and recently shifted to the mountainous region. She had poor clothing and was unaware of the risk of frostbite injury and the means to protect herself, using snow to relieve her pain due to frostbite, thus further aggravating her injury.
Zhang et al., 2022, China	▪ 36 patient cases were included in this retrospective case-series ▪ 17 (47%) of the 36 cases required amputation ▪ Frostbite risk factors were as follows for the cases: alcohol consumption (47.2%), mental disorder (22.2%), homelessness (11.1%), less cold weather protection (8.3%), outdoor sports (8.3%), and drug use (2.8%).
Zhao et al., 2020, China	▪ 156 patient cases were included in this retrospective case-series ▪ There were no statistically significant differences regarding age or gender for patients who required amputation and those who did not ▪ Significant factors for an increased amputation rate were delayed presentation to the hospital and a longer time period before rewarming (p-value = 0.004) ▪ Smoking and alcohol abuse were identified as predisposing factors for frostbite-related amputation (p-value < 0.001)

**Table 4:** Key and significant findings associated with frostbite-related injury

Authors, year, country	Main findings
Ervasti et al., 2004, Finland	▪ This cross-sectional study had a sample size of 5893 ▪ The lifetime occurrence of frostbite in the population was 44%, with a high occurrence among young and healthy individuals ▪ Smoking was found to be associated with frostbite injury
Ghumman et al., 2019, Canada	▪ 22 patient cases were included in this retrospective case-series ▪ Out of the total 22 patients, 4 patients (18%) required amputation due to frostbite injury ▪ Almost half of the patients (45.5%) were intoxicated (alcohol/drugs) during the time of injury ▪ Psychiatric illness (40.9%) was reported among 9 of the patients at the time of the injury
Hanko et al., 2022, USA	▪ 41 patient cases were included in this retrospective case-series ▪ The characteristics of the 41 subjects with frostbite injury were as follows: male (70%), homeless (39%), and substance use diagnosis (51.2%) ▪ Frostbite vulnerability was heightened by such risk factors as intoxication, psychosis-impaired judgment, and homelessness
Harirchi et al., 2005, Iran	▪ This cross-sectional study had a sample size of 637 mountaineers ▪ Out of the 637 mountaineers, 467 (73%) reported incidents of frostbite during the preceding 2 years ▪ 56% of the frostbite injuries were attributed to the inappropriate wearing of equipment (OR 14.3) ▪ Other major determinants of frostbite injury were inappropriate clothing, wet clothing, and lack of knowledge about dealing with cold and severe cold weather
Lehmuskallio et al., 1995, Finland	▪ This prospective case-control study had a sample size of 913 ▪ Risk factors for frostbite included not wearing proper winter apparel, being transported in open vehicles, applying emollients to the face and ear, being sensitive to the cold, and having hands and feet sweat profusely.
Makinen et al., 2009, Finland	▪ This cross-sectional study had a sample size of 13,713 ▪ Alcohol consumption, whether heavy or light, was associated with an increase in frostbite injury risk ▪ Respondents who had often felt depressed (psychiatric illness) showed a 2-3x higher OR for severe frostbite injury than those who did not
Masood et al., 2008, Pakistan	▪ This cross-sectional study had a sample size of 130 ▪ There was a significant relationship between lack of proper equipment or lack of knowledge and frostbite injury ▪ Defective snow gloves and high-altitude clothing led to 23.8% soldiers frostbite cases

## Study quality assessment

A combination of tools was used to assess the quality of included studies in the systematic review. The quality of observational studies, such as study selection, comparability and outcome measures were assessed on the Newcastle-Ottawa Scale (NOS) (Wells et al. 2014). The adapted NOS employs a star system where each star is assigned 1 point and a total score of 10 points, indicating a high-quality study with a lower risk of bias (Wells et al. 2014). Stars were assigned across three subscales: (1) Selection was assigned five stars and evaluated sample representativeness, size, non-response rate, and methods for assessing the exposure variable; (2) Comparability (two stars) addresses the control of confounding factors; and (3) Outcomes was assigned three stars and assessed the outcome variable, proper use and reporting of statistical tests. The total scores were grouped into four levels of methodological quality: unsatisfactory studies (0–4 points), satisfactory studies (5–6 points), good studies (7–8 points), and very good studies (9–10 points) (Wells et al. 2014). Detailed quality ratings for each study can be found in Table 5. In addition, the methodological quality of the included case reports and case series was assessed using the Murad et al. tool (Murad et al. 2018). The present study quantifies a study’s methodological quality as satisfactory if it accounted for at least five of the seven questions that applied to the present review and unsatisfactory if it accounted for four or fewer questions (Table 6).

**Table 5:** Methodological quality assessment of included cross-sectional, case-control and cohort studies (*n* = 11) using the Newcastle-Ottawa Scale

Author(s), year, country	Selection				Comparability	Outcome		Total score
	Representativeness of the sample (one star)	Sample size (one star)	Non-respondents (one star)	Ascertainment of the exposure (two stars)	Based on design and analysis (two stars)	Assessment of outcome (two stars)	Statistical test (one star)	
Endorf et al., 2022a, USA	*			**	**	**	*	8
Endorf et al., 2022b, USA	*	*		**	**	**	*	9
Endorf et al., 2021a, USA	*	*	*	**	**	**	*	10
Endorf et al., 2021b, USA	*	*	*	**	**	**	*	10
Ervasti et al., 2004, Finland	*	*	*	**	*	**	*	9
Fabian et al., 2017, Canada		*		**	*	**	*	7
Harirchi et al., 2005, Iran		*		*	*	**		5
Lehmuskallio et al., 1995, Finland		*		**	*	**	*	7
Lindford et al., 2017, Finland	*		*	**	**	**		8
Makinen et al., 2009, Finland	*	*		*	*	**	*	7
Masood et al., 2008, Pakistan		*	*	**	*	**		7

One star (✱) = 1 point. Quality score classification: 9–10 points = *very good studies*; 7–8 points = *good studies*; 5–6 points = *satisfactory studies*; 0–4 points = *unsatisfactory studies*

**Table 6:** Methodological quality assessment of included case reports and case series (*n* = 25)

Author(s), year, country	Selection	Ascertainment		Causality				Reporting sufficient details	Total score
	Patients’ representation	Exposure adequately ascertained	Outcome adequately ascertained	Alternative causes ruled out	Challenge/re-challenge phenomenon	Dose-response effect	Long enough follow-up		
Antti-Poika et al., 1990, Finland	1	1	1	1	0	1	0	1	6
Boles et al., 2018, Canada	1	1	1	1	0	1	0	1	6
Brändström et al., 2014, Sweden	1	1	1	1	0	1	0	1	6
Carceller et al., 2019, Spain	0	0	0	1	0	1	0	1	3
Cauchy et al., 2001, France	1	1	1	1	0	1	0	1	6
Detanac et al., 2022, Serbia	1	1	1	1	0	1	0	1	6
Ghumman et al., 2019, Canada	1	1	1	1	0	1	0	1	6
Hanko et al., 2022, USA	1	1	1	1	0	1	0	1	6
Hashmi et al., 1998, Pakistan	1	1	1	1	0	1	0	1	6
Jovic et al., 2019, Serbia	1	1	1	1	0	1	0	1	6
Kloeters et al., 2011, Germany	0	1	1	1	0	1	0	1	5
Koljonen et al., 2004, Finland	1	1	1	1	0	1	0	1	6
Lorentzen and Penninga, 2018, Greenland	0	1	1	1	0	1	0	1	5
Miller et al., 1980, Canada	0	1	1	1	0	1	0	0	4
Nygaard et al., 2017, USA	1	1	1	1	0	1	0	1	6
Poole et al., 2021, Canada	1	1	1	1	0	1	0	1	6
Schellenberg et al., 2020, USA	1	1	1	1	0	1	0	1	6
Su et al., 2015, China	1	1	1	1	0	1	0	1	6
Tavri et al., 2016, USA	1	1	1	1	0	1	0	1	6
Tran et al., 2022, USA	1	1	1	1	0	1	0	1	6
Urschel, J., 1990, Canada	1	1	1	1	0	1	0	0	5
Valnicek et al., 1993, Canada	1	1	1	1	0	1	0	1	6
Wani et al., 2008, India	0	1	1	1	0	1	0	1	5
Zhang et al., 2022, China	1	1	1	1	0	1	0	1	6
Zhao et al., 2020, China	1	1	1	1	0	1	0	1	6

Quality score classification: ≥5 points = *satisfactory studies*; 0–4 points = *unsatisfactory studies*

## Results

Of the 36 studies identified, 29 studies reported on psychosocial and personal predisposing factors of frostbite injuries that led to amputation, whereas seven studies reported on frostbite injuries that did not require amputation.

## Studies that reported frostbite injury that led to amputation

Of the 29 studies that reported on psychosocial and personal predisposing factors of frostbite injuries that led to amputation, three studies did not report on sex; however, one of these studies had only abstract available, four studies included only males, one reported on a female and the remaining studies included both males and females. Also, 25 of the 29 studies reported the participants’ age/mean or median age, with approximate ages/mean or median ages ranging from 15 to 60 years. The included studies employed different designs to explore psychosocial and personal predisposing factors of frostbite-related amputation. Most of the studies reviewed were retrospective case series (19 studies), six were retrospective cohort studies, and four were case reports. Further, a review of the 29 studies for psychosocial and personal predisposing factors of frostbite-related amputation revealed that 10 studies reported on homelessness, six studies on smoking, 21 studies on alcohol intoxication/abuse, 15 studies on psychiatric disorders, eight studies on substance use and one each reported on low socioeconomic status and unsupervised care.

Eight studies reported inadequate or improper winter clothing as a personal predisposing factor for frostbite-related amputations. Seven studies reported delays in seeking medical treatment, three on time before rewarming, and one each cited an individual’s higher admission heart rate and lack of knowledge of how to deal with cold weather as a personal factor influencing the progression from frostbite injuries to frostbite-related amputations. Moreover, of the 29 studies, amputation cases reported in 10 of the studies were due to deep frostbite injuries, both superficial and deep frostbite injuries led to amputation cases in 16 of the studies, and three studies did not report the severity of frostbite injury; however, two of these studies had only abstract available.

Twenty (Ghumman et al. 2019) studies looked at both the upper extremity (UE) and lower extremity (LE) frostbite-associated limb amputation, while three studies limited their data to UE or LE only. Six studies did not specify the extremity of amputation; however, one of these studies had only an abstract available. Studies included span across three continents, with the majority (15 studies) from North America (Canada:6, United States:8 and Greenland:1), followed by Europe with nine studies (Finland:3, Serbia:2, Germany:1, Sweden:1, France:1 and Spain:1) and five studies from Asia (China:3, India:1 and Pakistan:1).

## Studies that reported frostbite injuries that did not require amputation

Of the seven studies that reported frostbite injuries that did not require amputation, two reported on both males and females, three on males only, and the other two did not report on sex. The age/mean age of study participants was reported in five of the studies, ranging from 19 to 40 years, and two studies did not report age. Case series and cross-sectional study designs constituted most of the included studies, with two retrospective case-series studies, four cross-sectional studies and one prospective case-control study. The range of psychosocial and personal predisposing factors of frostbite injury reported in the included studies was notable. One study reported on homelessness, one on smoking, two studies reported on alcohol intoxication/abuse, three studies on psychiatric disorders, two studies on substance use, two each on inadequate or improper winter clothing, lack or incorrect use of equipment and lack of knowledge of how to deal with cold weather; Six studies reported both superficial and deep frostbite injuries, and one reported only superficial frostbite injury as severity of injury. Three of the studies were from Finland, and one each was from Canada, the United States, Iran, and Pakistan.

### Summary of findings on psychosocial and personal predisposing factors of included studies

Further insight into psychosocial and personal predisposing factors of frostbite injuries and associated amputations identified in the present review and presented in Tables 1 and 2 have been summarized and organized below.

### Psychosocial factors

Of the 36 studies included in the present review, 28 studies reported six psychosocial factors as major drivers of frostbite injuries and related amputation (Zhang et al. 2022; Ghumman et al. 2019; Urschel 1990; Endorf et al. 2022a; Fabian et al. 2017; Antti-Poika et al. 1990; Boles et al. 2018; Brändström et al. 2014; Cauchy et al. 2001; Detanac et al. 2022; Endorf and Nygaard 2021a, b, 2022b; Jovic et al. 2019; Koljonen et al. 2004; Lindford et al. 2017; Lorentzen and Penninga 2018; Miller and Chasmar 1980; Nygaard et al. 2017; Poole et al. 2021; Su et al. 2015; Tavri et al. 2016; Tran et al. 2022; Zhao at al. 2020; Ervasti et al. 2004; Hanko et al. 2022; Makinen et al. 2009; Wani et al. 2008). These factors were homelessness, alcohol intoxication/abuse, psychiatric disorders, substance use, smoking and low socioeconomic status. Alcohol intoxication/abuse was the most reported psychosocial factor, with 23 out of the 36 studies reporting alcohol intoxication/abuse as a predisposing factor for frostbite injuries and associated amputation (Zhang et al. 2022; Ghumman et al. 2019; Urschel 1990; Endorf et al. 2022a;  Fabian et al. 2017; Antti-Poika et al. 1990; Brändström et al. 2014; Cauchy et al. 2001; Endorf and Nygaard 2021a, b; Jovic et al. 2019; Koljonen et al. 2004; Lindford et al. 2017; Lorentzen and Penninga 2018; Miller and Chasmar 1980; Nygaard et al. 2017; Poole et al. 2021; Su et al. 2015; Tavri et al. 2016; Tran et al. 2022; Zhao et al. 2020; Makinen et al. 2009; Wani et al. 2008), followed by a psychiatric disorder, reported in 18 out of the 36 studies reviewed (Zhang et al. 2022; Ghumman et al. 2019; Urschel 1990; Fabian et al. 2017; Boles et al. 2018; Cauchy et al. 2001; Detanac et al. 2022; Endorf and Nygaard 2021a, b; Jovic et al. 2019; Koljonen et al. 2004; Lindford et al. 2017; Lorentzen and Penninga 2018; Nygaard et al. 2017; Su et al. 2015; Tran et al. 2022; Hanko et al. 2022; Makinen et al. 2009). Homelessness emerged third, with 11 studies (Zhang et al. 2022; Endorf et al. 2022a; Detanac et al. 2022; Endorf and Nygaard 2021a, b, Endorf et al. 2022a; Jovic et al. 2019; Nygaard et al. 2017; Tavri et al. 2016; Tran et al. 2022; Hanko et al. 2022) out of the 36 studies reporting a relationship between homelessness and frostbite injuries and associated amputation. Only six studies reported on all three factors: alcohol intoxication/abuse, psychiatric disorder and homelessness (Zhang et al. 2022; Endorf and Nygaard 2021a, b; Jovic et al. 2019; Nygaard et al. 2017; Tran et al. 2022), whereas the remaining reported on either one or two of the factors.

Eight out of the 11 studies that reported on homelessness came from the United States (Endorf et al. 2022a;  Endorf and Nygaard 2021a, b, 2022b; Nygaard et al. 2017; Tavri et al. 2016; Tran et al. 2022; Hanko et al. 2022), and only three of the studies quantified the association between homelessness and frostbite injuries/frostbite-associated amputation with odds ratios (Endorf and Nygaard 2021a, 2022b; Endorf et al. 2022a). According to the three studies, homeless individuals were 5.40, 1.62 and 1.81 more likely to sustain frostbite-related amputation (Endorf et al. 2021a, 2022b; Endorf et al. 2022a). The remaining studies were based on descriptive statistics (e.g., number of cases, proportions, and p-values from Chi-square) (Zhang et al. 2022; Detanac et al. 2022; Endorf and Nygaard b; Jovic et al. 2019; Nygaard et al. 2017; Tavri et al. 2016; Tran et al. 2022; Hanko et al. 2022).

Moreover, substance use was reported by 10 studies (Zhang et al. 2022; Ghumman et al. 2019; Endorf et al. 2022a; Boles et al. 2018; Endorf and Nygaard 2021a, b; Lindford et al. 2017; Nygaard et al. 2017; Tran et al. 2022; Hanko et al. 2022), with the magnitude of association quantified as odds ratios in one of the studies (Endorf et al. 2022a). According to the study, persons with substance use disorder were 3.19 times more likely to sustain frostbite-related amputation than their counterparts with no substance use disorder (Endorf et al. 2022a). Smoking was reported by seven studies (Zhang et al. 2022; Boles et al. 2018; Jovic et al. 2019; Lindford et al. 2017; Tavri et al. 2016; Zhao et al. 2020; Ervasti et al. 2004) and low socioeconomic status (Wani et al. 2008) was reported by one as a predisposing psychosocial factor associated with frostbite injuries and related amputation.

### Personal factors

Out of the 36 studies, 16 reported personal predisposing factors, including delay in seeking medical care, time before rewarming, lack of knowledge of how to deal with the cold, and inadequate/improper winter clothing. Seven of the 16 studies identified delays before receiving medical treatment/attention as a personal predisposing factor associated with frostbite injuries and related amputation (Carceller et al. 2019; Urschel 1990; Nygaard et al. 2017; Zhao et al. 2020; Wani et al. 2008; Kloeters et al. 2011; Valnicek et al. 1993). Furthermore, three studies identified time before rewarming (Carceller et al. 2019; Nygaard et al. 2017; Zhao et al. 2020), and one study reported an individual’s higher admission heart rate as a personal factor influencing the progression from frostbite injuries to frostbite-related amputations (Schellenberg et al. 2020). Inadequate or improper winter clothing was reported by 10 studies (Zhang et al. 2022; Fabian et al. 2017; Cauchy et al. 2001; Detanac et al. 2022; Lorentzen and Penninga 2018; Wani et al. 2008; Valnicek et al. 1993; Hashmi et al. 1998; Harirchi et al. 2005; Lehmuskallio et al. 1995), whereas lack/incorrect equipment use was reported by two studies (Harirchi et al. 2005; Masood et al. 2008), and knowledge of how to deal with cold weather was reported by three (Wani et al. 2008; Harirchi et al. 2005; Masood et al. 2008) studies as predisposing factors for frostbite injuries.

### Quality assessments

The risk of bias assessment of included studies is summarized in Table 5. Eleven (Whitfield 2024) studies were assessed using the Newcastle-Ottawa Scale (Endorf and Nygaard 2021a, b, 2022b; Endorf et al. 2022a; Fabian et al. 2017; Lindford et al. 2017; Ervasti et al. 2004; Makinen et al. 2009; Harirchi et al. 2005; Lehmuskallio et al. 1995; Masood et al. 2008). Four studies were classified as very good based on the scale, of which three were retrospective cohort studies (Endorf and Nygaard 2021a, b, 2022b), and one was a cross-sectional study (Ervasti et al. 2004). Six studies (Endorf et al. 2022a; Fabian et al. 2017; Lindford et al. 2017; Makinen et al. 2009; Lehmuskallio et al. 1995; Masood et al. 2008) were classified as good studies by the Newcastle-Ottawa Scale, of which three were retrospective cohort studies (Endorf et al. 2022a; Fabian et al. 2017; Lindford et al. 2017), two were cross-sectional studies (Makinen et al. 2009; Masood et al. 2008), and one was prospective case-control (Lehmuskallio et al. 1995). According to the Newcastle-Ottawa Scale, only one study was classified as satisfactory (Harirchi et al. 2005). Similarly, the quality assessment of 25 studies based on the Murad et al. tool and presented in Table6, of which four were case reports (Detanac et al. 2022; Lorentzen and Penninga 2018; Wani et al. 2008; Kloeters et al. 2011), and 21 were case series (Carceller et al. 2019; Zhang et al. 2022; Ghumman et al. 2019; Urschel 1990; Antti-Poika et al. 1990; Boles et al. 2018; Brändström et al. 2014; Cauchy et al. 2001; Jovic et al. 2019; Koljonen et al. 2004; Miller and Chasmar 1980; Nygaard et al. 2017; Poole et al. 2021; Su et al. 2015; Tavri et al. 2016; Tran et al. 2022; Zhao et al. 2020; Hanko et al. 2022; Valnicek et al. 1993; Nygaard et al. 2017; Poole et al. 2021; Su et al. 2015; Tavri et al. 2016; Tran et al. 2022; Hashmi et al. 1998), revealing that the majority of the studies (23 studies) methodological quality were satisfactory based on at least five of the seven questions that applied to the included case reports and case series studies (Zhang et al. 2022; Ghumman et al. 2019; Urschel 1990; Antti-Poika et al. 1990; Boles et al. 2018; Brändström et al. 2014; Cauchy et al. 2001; Detanac et al. 2022; Jovic et al. 2019; Koljonen et al. 2004; Lorentzen and Penninga 2018; Nygaard et al. 2017; Poole et al. 2021; Su et al. 2015; Tavri et al. 2016; Tran et al. 2022; Zhao et al. 2020; Hanko et al. 2022; Wani et al. 2008; Kloeters et al. 2011; Valnicek et al. 1993; Schellenberg et al. 2020; Hashmi et al. 1998). Only two studies’ methodological quality was unsatisfactory based on four or fewer questions (Carceller et al. 2019; Miller and Chasmar 1980) of the seven questions.

## Discussion

The present review identified 36 articles specifically focused on psychosocial and personal predisposing factors of frostbite injuries and associated amputations. Twenty-nine studies reported on predisposing factors of frostbite injuries that led to amputation (Carceller et al. 2019; Zhang et al. 2022; Urschel 1990; Endorf et al. 2022a; Fabian et al. 2017; Antti-Poika et al. 1990; Boles et al. 2018; Brändström et al. 2014; Cauchy et al. 2001; Detanac et al. 2022; Endorf and Nygaard 2021a, b, 2022; Jovic et al. 2019; Koljonen et al. 2004; Lindford et al. 2017; Lorentzen and Penninga 2018; Miller and Chasmar 1980; Nygaard et al. 2017; Poole et al. 2021; Su et al. 2015; Tavri et al. 2016; Tran et al. 2022; Zhao et al. 2020; Wani et al. 2008; Kloeters et al. 2011; Valnicek et al. 1993; Schellenberg et al. 2020; Hashmi et al. 1998), whereas seven studies reported on frostbite injuries that did not require amputation (Ghumman et al. 2019; Ervasti et al. 2004; Hanko et al. 2022; Makinen et al. 2009; Harirchi et al. 2005; Lehmuskallio et al. 1995; Masood et al. 2008). Identical psychosocial and personal predisposing factors for frostbite injuries and frostbite-related amputations were reported in most of the studies reviewed. Six psychosocial predisposing factors were observed in 28 out of the 36 studies reviewed: homelessness, psychiatric disorders, substance use, alcohol intoxication/abuse, smoking and low socioeconomic status. While each of these psychosocial factors may independently increase the risk of frostbite injuries and associated amputation, they frequently co-occur, particularly among homeless individuals, who exhibit high rates of psychiatric disorders, alcohol intoxication/abuse, substance use disorders and smoking (Martens 2001; Ayano et al. 2019). Most studies on homelessness in the present review came from the United States (Endorf and Nygaard 2021a, b, 2022b; Endorf et al. 2022a; Nygaard et al. 2017; Tavri et al. 2016; Tran et al. 2022; Hanko et al. 2022), which was not surprising because the United States has one of the highest numbers of homelessness in the world and for that matter, in the cold regions of the world (Lihanceanu 2024; Casey and Stazen 2021). Reports from the United States indicate that one-third of homeless individuals suffer from severe mental health illness (Homelessness Treatment Advocacy Center 2023), and they are at higher risk for cold-weather injuries, including frostbite, when compared to the general population (National Coalition for the Homeless 2014). This review confirms that psychiatric disorders found significantly contribute to the risk factor of frostbite and related amputations (Zhang et al. 2022; Ghumman et al. 2019; Urschel 1990; Fabian et al. 2017; Boles et al. 2018; Cauchy et al. 2001;  Detanac et al. 2022; Endorf and Nygaard 2021a, b; Jovic et al. 2019;  Koljonen et al. 2004; Lindford et al. 2017; Lorentzen and Penninga 2018; Nygaard et al. 2017; Su et al. 2015; Tran et al. 2022; Hanko et al. 2022; Makinen et al. 2009), aligning with previous findings from Reamy’s extensive review of frostbite, which also highlighted the association between psychiatric illness and frostbite (Reamy 1998). In contrast, Endorf et al., whose study was cited in this review, found that psychiatric diagnosis unrelated to substance use was not associated with amputation in frostbite patients (Endorf et al. 2022a). Although several studies in the present review noted an association between substance use and frostbite injuries and related amputation (Zhang et al. 2022; Ghumman et al. 2019; Endorf and Nygaard 2021a, b; Endorf et al. 2022a; Boles et al. 2018; Lindford et al. 2017; Nygaard et al. 2017; Tran et al. 2022; Hanko et al. 2022), the relationship between substance use and psychiatric diagnoses remains unclear. The established co-occurrence of psychiatric disorders and substance use disorders, established in other published reviews (Ross et al. 2012; Bahji 2024; Kingston et al. 2017), suggests a complex interplay that warrants further investigation to better understand the differential impact of psychiatric diagnoses, both those related and unrelated to substance use, on frostbite and related amputations.

The findings of this review indicating that alcohol abuse/intoxication are major contributing/etiologic factors to frostbite and related amputation are consistent with those identified in Reamy’s earlier review (Reamy 1998). Moreover, evidence suggests that nicotine, including from smoking, can lead to vasoconstriction of skin blood vessels (Ervasti et al. 2004; Black et al. 2001; Defense Centers for Public Health 2024), which is linked to an increased risk of frostbite (Defense Centers for Public Health 2024). This proposed mechanism may explain why smoking is identified as a risk factor for frostbite and related amputations (Zhang et al. 2022; Boles et al. 2018; Jovic et al. 2019; Lindford et al. 2017; Tavri et al. 2016; Zhao et al. 2020; Ervasti et al. 2004).

Similarities and differences were observed in personal predisposing factors for frostbite-related amputation studies and frostbite injuries that did not require amputation studies. Whereas delay before receiving medical attention (Carceller et al. 2019; Urschel 1990; Nygaard et al. 2017; Zhao et al. 2020; Wani et al. 2008; Kloeters et al. 2011; Valnicek et al. 1993), the time before rewarming (Carceller et al. 2019; Nygaard et al. 2017; Zhao et al. 2020) and an individual’s higher admission heart rate (Schellenberg et al. 2020) were predisposing factors for frostbite-related amputation, lack or incorrect use of equipment was found to be associated with only frostbite injury (Harirchi et al. 2005; Masood et al. 2008). Inadequate or improper winter clothing (Zhang et al. 2022; Fabian et al. 2017; Cauchy et al. 2001; Detanac et al. 2022; Lorentzen and Penninga 2018; Wani et al. 2008; Valnicek et al. 1993; Hashmi et al. 1998; Harirchi et al. 2005; Lehmuskallio et al. 1995), and lack of knowledge of how to deal with cold weather (Wani et al. 2008; Harirchi et al. 2005; Masood et al. 2008) were identified as predisposing factors for both frostbite injuries and associated amputation. The former and latter findings are attributable to low socioeconomic status (Wani et al. 2008). Wani et al.‘s case report study, cited in the present review, noted that low socioeconomic status deprived the patient of education, led to a lack of knowledge about dealing with cold weather conditions and failure to recognize that the injury resulted from frostbite and as well impacted the patient’s ability to afford proper winter clothing (Wani et al. 2008). Although better education to increase people’s knowledge about cold weather protection (Endorf and Nygaard 2021b; Hall et al. 2018), especially in homeless individuals, could help reduce the incidence of frostbite injuries and associated limb loss, some homeless individuals are knowledgeable about cold weather and proper winter clothing, but the affordability of protective gears is a challenge, especially in the face of the rising cost of living escalating affordability crisis globally (The British Psychological Society 2024), which is forcing many people into homelessness (Heston 2023) and exacerbating the suffering of vulnerable populations including those who are already homeless (The British Psychological Society 2024). Hence, directing more resources toward providing housing where possible and warm protective gear to homeless individuals could further reduce frostbite injuries and associated limb loss.

Not all frostbite injuries lead to devasting outcomes, as some injuries are mild (Gupta et al. 2021). However, as the severity of frostbite increases, dependent on multiple factors, including the duration and intensity of cold exposure, it can result in limb loss (Carceller et al. 2019; Ikäheimo et al. 2011). Factors that contribute to prolonged cold exposure include delays in receiving medical care or rewarming (Carceller et al. 2019; Urschel 1990; Nygaard et al. 2017; Zhao et al. 2020; Wani et al. 2008; Kloeters et al. 2011; Valnicek et al. 1993), which may be considered personal predisposing factors. However, these delays can also be attributed to a lack of/inadequate emergency response services in unforeseen circumstances, such as motor vehicle accidents during a cold winter season, particularly in rural or less motorable highways, as highlighted in one of the studies reviewed (Kloeters et al. 2011).

Furthermore, while most studies on frostbite-related amputation report an age/mean age of 40 years and older (Zhang et al. 2022; Fabian et al. 2017; Antti-Poika et al. 1990; Brändström et al. 2014; Detanac et al. 2022; Koljonen et al. 2004; Lorentzen and Penninga 2018; Su et al. 2015; Valnicek et al. 1993; Schellenberg et al. 2020), studies describing frostbite injuries that did not require amputation reported an age/mean age below 40 years (Ervasti et al. 2004; Hashmi et al. 1998; Harirchi et al. 2005; Lehmuskallio et al. 1995). This suggests that advanced age may influence the progression of frostbite injury to frostbite-related amputation, a notion supported by Nygaard et al., who found that older frostbite victims tended to require amputation (Nygaard et al. 2017). In addition, the present review identified more frostbite injuries and associated limb amputation in males compared to females. The evidence that females are less impacted by frostbite injury and it’s devasting effects is supported by a previous review that examined frostbite-related mortality in mountaineering women and found that the risk of frostbite mortality was lower in females than in males but concluded that the sex differences observed in frostbite were inconclusive (Kriemler et al. 2023).

However, the observed sex difference highlighted in this review may, in part, stem from the dominance of males in most frostbite-related outdoor sports and occupations, such as military service (Lehmuskallio et al. 1995), mountaineering (Frohlick 1999) and fishing (Lorentzen and Penninga 2018). Furthermore, the sex difference can be contextualized by the varying psychosocial predisposing factors that affect males and females. For example, the present review identified homelessness as one of the leading psychosocial predisposing factors for frostbite injury and associated limb amputation. It is important to note that homelessness disproportionately impacts genders; research by Moses and Janosko’s identified that 70% of homeless individuals are men, 29% are women, and the remaining 1% identify as transgender or non-binary in America (Moses and Janosko 2018).

### Methodological considerations of included studies

The strong evidence of the association between predisposing factors and frostbite injuries or frostbite-related amputations observed in the studies reviewed is supported by the robust study designs and unbiased assessment of the study outcomes (frostbite injuries or frostbite-related amputations), which is a direct surgical intervention devoid of reporting or recall bias. Although case series are generally prone to the risk of bias (Murad et al. 2018), Murad et al. reiterated the need to include case series in reviews for evidence-based decision-making, especially in the absence of study designs with higher levels of evidence (Murad et al. 2018).

### Strengths and limitations

The review provides comprehensive information on psychosocial and personal factors predisposing people to frostbite injury and associated limb amputation. This review had a few limitations. Most studies included in the present review were case series known for their elevated risk of bias (Murad et al. 2018). Also, heterogeneity in the methods used to quantify the measure of association in the included studies, where some were descriptive and other inferential tests, precluded the present review from further pursuing meta-analysis. Due to the cross-sectional nature of some of the studies, especially those that relied on only administrative data may not capture whether early detected frostbite cases later resulted in amputation as, in most cases, the tissue must be allowed to demarcate before amputation is preformed (Paine et al. 2020; Carceller et al. 2017). Few studies had only published abstracts available, with limited details for extraction (Urschel 1990).

## Conclusions

While more resources and studies must focus on ways to better care for frostbite injuries and associated limb loss, it is equally important to direct efforts toward mitigating the psychosocial predisposing factors of frostbite injuries and associated limb amputations. This need is particularly urgent in some of the coldest regions in the world, where rising rates of homelessness, compounded by a disproportionality higher rate of substance and alcohol abuse McVicar et al. (2015), could potentially predispose people to frostbite and limb amputation, as evidenced by the present review, where homelessness, substance use and alcohol intoxication/abuse were identified as leading predisposing factors of frostbite injuries and associated limb amputation.

## Data Availability

No datasets were generated or analysed during the current study.
